# On the Influence of Infra-Red Sensor in the Accurate Estimation of Grinding Temperatures

**DOI:** 10.3390/s18124134

**Published:** 2018-11-26

**Authors:** Lander Urgoiti, David Barrenetxea, Jose Antonio Sánchez, Iñigo Pombo, Jorge Álvarez

**Affiliations:** 1 IK4-IDEKO Research Alliance, Arriaga Kalea, 2, 20870 Elgoibar, Spain; dbarrenetxea@ideko.es (D.B.); jalvarez@ideko.es (J.Á.); 2 Mechanical Engineering Department, Universidad del País Vasco (UPV/EHU), Paseo Rafael Moreno “Pitxitxi”, 3, 48013 Bilbao, Spain; joseantonio.sanchez@ehu.eus (J.A.S.); inigo.pombo@ehu.eus (I.P.); 3 Aeronautics Advanced Manufacturing Center (CFAA), University of the Basque Country (UPV/EHU), 48170 Zamudio, Spain

**Keywords:** grinding, temperatures, pyrometry, dichromatic photodiode

## Abstract

Workpiece rejection originated by thermal damage is of great concern in high added-value industries, such as automotive or aerospace. Surface temperature control is vital to avoid this kind of damage. Difficulties in empirical measurement of surface temperatures in-process imply the measurement in points other than the ground surface. Indirect estimation of temperatures demands the use of thermal models. Among the numerous temperature measuring techniques, infra-red measurement devices excel for their speed and accurate measurements. With all of this in mind, the current work presents a novel temperature estimation system, capable of accurate measurements below the surface as well as correct interpretation and estimation of temperatures. The estimation system was validated by using a series of tests in different grinding conditions that confirm the hypotheses of the error made when measuring temperatures in the workpiece below the surface in grinding. This method provides a flexible and precise way of estimating surface temperatures in grinding processes and has shown to reduce measurement error by up to 60%.

## 1. Introduction

Grinding is a key technology for high-added value sectors including aerospace, automotive, and energy generation [1]. Grinding allows for the machining of hardened or difficult-to-machine materials, leaving an excellent geometric tolerance and surface finish. In many cases, grinding is the last step during a complete manufacturing process for a given component. This means that, after certain cost and time-consuming preliminary operations (such as pre-machining, heat treatment, and intermediate quality control) any problem during grinding may ruin the complete production chain. In fact, grinding is a complex process affected by several problems of varying nature. Recent publications show that vibrations [2], tool wear [3], and thermal damage [4] are all common causes of grinding problems. Such problems make grinding a field of interest for both academia and industry.

The concept of grinding burn is related to the thermal phenomena occurring during grinding operations. Malkin and Guo [5] have shown that, in a typical grinding operation, as much as 60–85% of the total consumed power is conducted to the workpiece in the form of heat. This results in a dramatic increase in workpiece temperature. Both temperature and contact time are key determinants of whether a given amount of heat causes thermal damage [6]. As a consequence, detrimental effects such as hardness decrease, whilst the generation of a white etching layer (WEL), surface cracks, oxidation, tensile residual stress, and others [7] may appear on the final component. 

The generation of large amounts of heat is related to the abrasive nature of the grinding process. In fact, shearing, as a main difference from other machining processes (such as turning or milling), does not play a predominant role in grinding. Instead, micro-chip generation, friction, and plastic deformation are predominant [8]. Due to this heating, temperature may rise on the workpiece surface to above 800 °C and local temperature gradients can be as high as 4000 °C/s during a typical surface grinding operation. In addition, the fact that grinding a non-defined cutting-edge machining operation means that the theoretical contact time and geometry may vary in the real process. These factors make it necessary to measure precisely the grinding temperatures, as well as the wheel and workpiece contact. When considering the presence of cooling fluid at high pressure, the actual temperature measurement in grinding is a very complex task.

A number of authors in the literature have proposed the use of thermocouples [7,8,9]. However, their accuracy is limited due to the thermal inertia of the device itself, and the reliance on the quality of the thermocouple-workpiece bond. Other temperature measuring methods are shown in [10] where Davies et al. made a state-of-the-art classification of the thermal measuring devices used and methods for material removal processes. Infra-red (IR) thermometry shows great potential for measuring temperatures in aggressive environments such as those related to machining processes [11,12].

The fundamentals of IR pyrometry are described in a NASA (National Aeronautics and Space Administration) technical note [13]. In this report, the basic laws and functioning mechanisms as well as extensive application details were presented. A complete description of the design and set-up of IR sensors and their application to conventional machining operations can be found in [14,15]. In the latter, IR pyrometry was applied by the authors to measure temperatures in the dry turning of Inconel 718. However, in the case of grinding, the already mentioned large temperature gradients and limited access to the heated spot make temperature measurement much more difficult.

The first studies to measure IR temperatures in a grinding operation were presented in the 1990s by Ueda et al. In [16], the authors used an optic fiber connected to a simple photodiode (monochromatic) to measure the temperature of the abrasive grits on the grinding wheel. The experiments were carried out under dry grinding conditions and the optic fiber was located in a fixed fiber holder just after the contact zone (ɸ = 45°). The objective was to analyze the effect of the cutting conditions on the temperature of the wheel. In a later study by the same authors [17], the temperature of a cutting grain was measured using a two-cell photodiode (dichromatic). Temperatures were obtained by comparing the two measurements of both cells, thus compensating the uncertainty caused by the emissivity of the material. This is known as ratio or two-color (dichromatic) pyrometry.

Regarding the position of the measuring probe, temperature measurements with pyrometers in grinding are rather difficult to make from the outside, since the large quantities of cutting fluid prevent access to the contact area. Brinksmeier et al. proposed the use of single color pyrometers installed in the grinding wheel [18]. Although this approach facilitates access to the contact area, it does not completely avoid the possibility that the presence of a cutting fluid film between wheel and workpiece could introduce uncertainty in the measurement. The alternative is to integrate the measuring probe inside the workpiece. In a more recent article, Reimers at al. [19] used an IR monochromatic sensor array placed inside an 18 mm hole at the side of the workpiece. The authors note that the sensor did not detect the heating period when using higher feed speeds, as occurs with lower feed speeds, leading to a variation between measurements. This is due to the distance between the measuring point and the ground surface. Moreover, the (measured) lateral conduction is minor to the thermal gradients in the workpiece depth axis, increasing the difference in temperature between the measured point and the ground surface. Therefore, this method of measurement has shown to be unreliable as well as incapable of measuring the contact. In this work, we propose to take the measurement below the ground surface, as this is believed to be the optimal positioning of the measurement probe to accurately capture the high temperature gradients that allow for a correct thermal characterization.

Measurements inside the workpiece demand the use of thermal models, since the measured point will not be exactly in the contact area. Furthermore, there will be a thermal distortion caused by the mere presence of a cavity in the workpiece. This latter effect was acknowledged in [20], where Xu et al. noticed a higher temperature than expected, explaining this in terms of the cavity. Although this effect alters the measured temperature, it has not yet been studied. Many authors use different models to support their measurements. The most recent modeling techniques used numerical solutions to solve the thermal equations. An example of these models can be found in [19] in which Reimers et al. used the Finite Element Method to model the thermal setup as well as the cavity and cutting fluid boundary conditions. As shown, infrared sensors show great potential for accurate temperature measurement in grinding operations. However, it is believed that the optimum measurement setup for exploiting the full potential of infrared sensors has not yet been achieved or proposed.

On the basis of the literature review it is clear that measuring surface and subsurface temperatures during industrial grinding operations it is still an issue to be resolved. For this reason, extensive research is still required on this topic. Therefore, this paper presents a novel approach to achieving efficient and accurate temperature estimation in grinding. The original approach involves the use of a high-precision two-color pyrometer together with optic fiber, which is combined with a thermal numerical model to cope with the distortion in the measurements due to the intrusive effect of the measuring instrument. This allows for an extremely accurate estimation of surface temperatures at points that could not be accessed by measuring devices. An explanation of the measurement device is provided in Section 2 of this work. Section 3 provides an explanation of the temperature estimation system as well as the setup of the experiments conducted to validate this system. The experimental results are presented and discussed in Section 4. Finally, conclusions are presented regarding the results obtained.

## 2. Pyrometer Design

Based on previous works, such as [14,15,17], a pyrometer that meets the needs of the grinding operation was designed. The pyrometer needed to be capable of measuring temperatures in the workpiece between 200 °C and 1100 °C. For this temperature interval, the sensor used was a sandwich architecture dichromatic InGaAs-InGaAs photodiode whose peak sensitivity wavelength were centered at 1.55 µm and 2.1 µm. The two cells of the photodiode were superposed one to the other and each reacts to a part of the wavelength range. 

**Figure 1:**
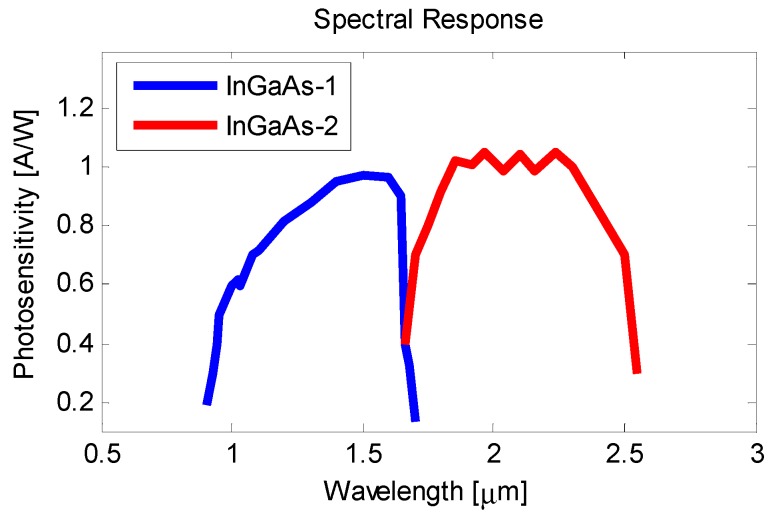
Spectral response of each of the cells that comprise the photodiode.

The high InGaAs cell, hereinafter called InGaAs-1, absorbs the shorter wavelengths between 0.9 µm and 1.7 µm, while the low InGaAs cell, hereinafter called InGaAs-2, reacts to longer wavelengths between 1.7 µm and 2.55 µm. The range of wavelengths allows the signal of both sensor cells to be strong enough within the mentioned temperature interval. Figure 1 displays the spectral response of both cells of the photodiode.

Temperature gradients measured in a static point located in the workpiece of a grinding operation can reach an order of magnitude of 4000 °C/s. To capture and outline such temperature changes, a rapid response sensor is essential. The sensors’ maximum 10 to 90 rise time is 200 ns. Acquisition frequency has been limited to reduce the excess of redundant data and facilitate the management and processing of the data. For this work, the data acquisition rate was set to 9000 Hz for each cell. The criterion of a minimum of 1200 measurement points on the smallest theoretical contact area was established. The authors considered this to be more than sufficient to depict temperature curves with such gradients.

The gray-body behavior is key to ratio pyrometry, and thus, certain considerations must be emphasized. This behavior occurs when the emissivity factor of the material is lower than 1 but still constant throughout the (measured) wavelength range. In [17], Ueda argued that as both ranges of the two monochromatic photodiodes are close to each other it is possible to assume that the emissivity is not dependent on wavelength, but constant. Given that the wavelength bands used in this paper are similar to those used by Ueda et al., the same behavior can be expected.

The radiation emitted by the cavity will be transported to the sensor by an optic fiber. This allows for more flexibility in the probe positioning due to the low level of intrusiveness of its small diameters. The selected fiber should be capable of transporting wavelengths within the sensitivity range of all the sensors. A low OH (Hydroxyl Groups) optic fiber was selected, since this fulfills the mentioned criterion and is available in a number of diameters. For the experiments conducted in this work, where cavities of Ø1 mm were used, a 300 µm diameter fiber was selected. This avoids the noise caused by a smaller fiber while the spot size does not exceed the size of the bottom of the cavity. 

Attached to the pyrometer, a self-developed data acquisition card was installed. This data acquisition card allows wireless control of the pyrometer as well as signal processing and management. With the use of this card, the pyrometer data capture can be switched on and off as well as setting the acquisition frequency. The card uses a 12 bit ADC converter. After each experiment was finished, the card stored the recorded data in a .txt file, ready for post-processing.

**Figure 2:**
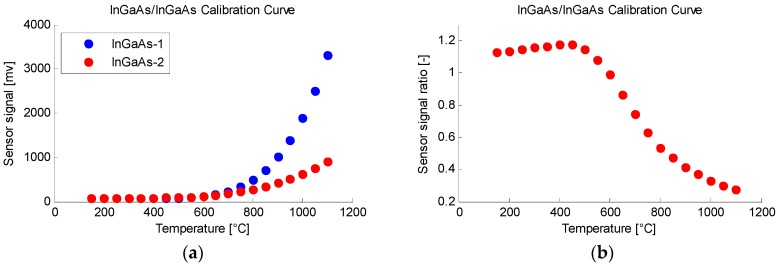
Experimental calibration points for the pyrometer presented in this work. (**a**) Represents the signal on each of the cells during the temperature rise. (**b**) Is the calibration curve obtained by dividing the values in both sensors.

A calibration was needed to overcome the possible measurement drifts caused by the assembly and to have an empirically tested calibration curve. For this, a calibration furnace was used. The diameter to length dimensions of the inside of the furnace simulate a blackbody that was heated to a certain stable temperature. Over the furnace, a tripod was placed to which an optic device was attached. With the use of the optic device it was possible to focus a 25 mm spot at a distance of 200 mm from the diameter of the fibers, thereby maintaining the optic fiber and the rest of the equipment at a safe distance from the intense heat that is emitted. Measurements were made from 150 °C to 1100 °C in 50 °C intervals (see Figure 2) with a stability of ±1 °C. The calibration points in Figure 2 depict a curve that represents the behavior of the pyrometer throughout the rises in temperature. From 200 to 450 °C the signal of InGaAs-2 cell was stronger than the signal of InGaAs-1 cell; thus, the ratio rises with the temperature. From 450 °C onward, the ratio falls as the temperature rises. This behavior is key to understanding the pyrometer readings, since a careless reading of the calibration can lead to a non-biunivocal response.

## 3. Materials and Methods

Two sets of grinding conditions were tested in holes machined on a GG-30 cast iron workpiece (see Table 1). The objective of the experiments was to validate the innovative temperature measurement system presented in this work and its ability to estimate the surface temperature in combination with the model. To this end, grinding experiments were conducted where different power and contact conditions were met, maintaining the removal rate or productivity using different combinations of depth of cut and feed rate. Consequently, contact length will change, whilst a change in the total power was also expected due to a change in kinematic conditions. Measurements of temperature and consumed power were taken during the process. In parallel, the same grinding, geometric, and thermal conditions were simulated for comparison with the measured results.

On each test, temperature was measured in two cavities at the same time with two identical pyrometers. Moreover, each set of conditions was tested in a different pair of cavities. Each measurement was labeled as S1 (Set 1) or S2 (Set 2), depending on the set of conditions used, and a numerical label was composed using the measurement number and the corresponding pyrometer. For instance, the test label S1-3.2 would be the first set of conditions measured on the third test by the pyrometer number two. Table 2 shows the real grinding conditions in which each measurement was made as well as the total consumed power that was measured.

**Figure 3:**
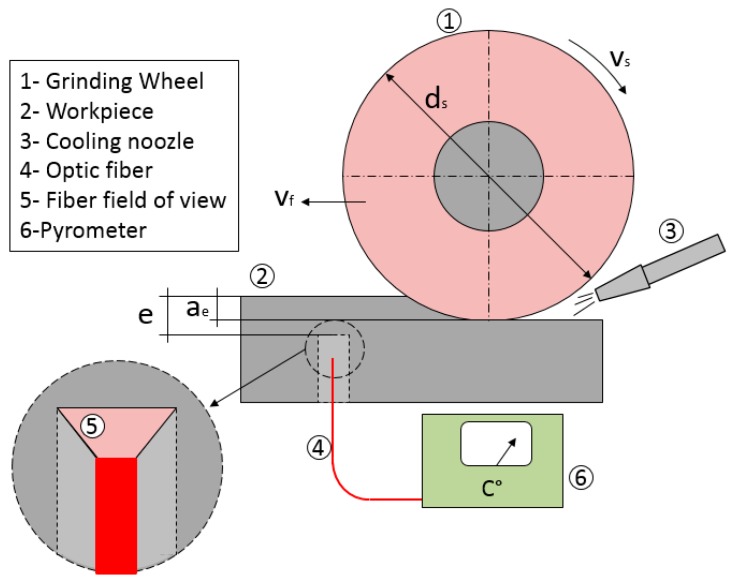
Experimental setup scheme.

In Figure 3, the full experimental assembly is shown, where $d_{s}$ is the grinding wheel diameter, $v_{f}$ the table feed rate, and $v_{s}$ is the grinding wheel peripheral speed. The workpiece thickness from the bottom of the cavity to the ground surface is represented as e and the real depth of cut as $a_{e}$. These two parameters must be carefully measured on every test. The exact distance between the cavity bottom and the ground surface in each pass must be known to precisely characterize the thermal modeling conditions. For this, at the end of the test, the workpiece was cut through the cavity center to measure the exact remaining distance from the bottom of the cavity to the surface. 

To achieve the best measurement conditions, the fiber used to measure spot distance should be carefully observed. The entirety of the bottom of the cavities covered by the fibers’ field of view should be optimal, and for this, the Numerical Aperture (NA = 0.39) of the fibers must be considered. For the Ø1 mm holes, the minimum distance from the fiber to the bottom of the cavity will be 0.85 mm.

As explained previously, the approach involved using the pyrometer together with a numerical thermal model in order to estimate grinding temperatures at the contact region between wheel and workpiece (see Figure 3). Therefore, a heat conduction model was also implemented.

**Figure 4:**
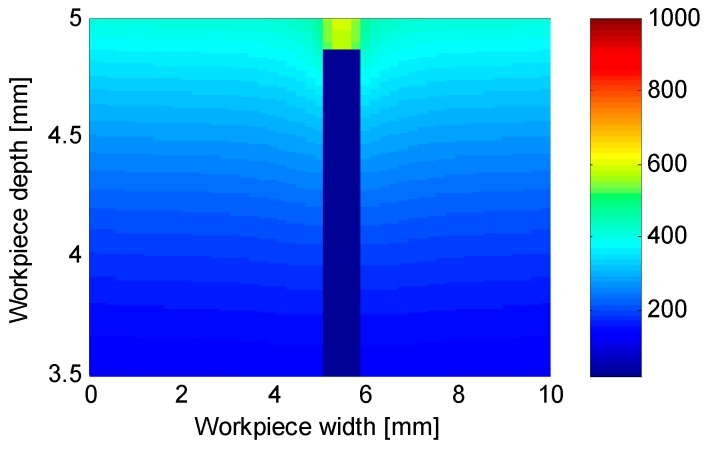
Temperature distribution in a section of the workpiece at the maximum temperature on the cavity bottom.

Since the measured spot was not on the ground surface, the experimental results needed to be interpreted. Moreover, the cavity needed for the measuring probe would cause a distortion in the temperature distribution [20]. An example of this distortion can be seen in Figure 4 where the temperature above the cavity is clearly higher.

The model uses a simple but nonetheless very powerful Finite Difference numerical method to assess the thermal processes involved. The thermal equations described in [21] were adapted to the grinding scenario in which the grinding thermal load, the effect of the cooling fluid, and the presence of the cavity were included. The grinding thermal load was modeled with the use of the conventional equation provided by Malkin et al. in [5]:

(1) $$\;q=\frac{\epsilon .P}{l_{c}.b}\;W/{\mathrm{mm}}^{2}\;$$

where q is the grinding heat per unit area penetrating in the workpiece, ϵ is the heat partition coefficient to the workpiece, P is the grinding power, $l_{c}$ is the length of cut, and b is the width of the ground surface.

As said, a convection boundary condition was included to simulate the flow of cutting fluid during the process. An experimental value of h = 2000 W/m^2^K was considered appropriated for this particular situation based on the work in [22] by Barrenetxea et al., where an experimental adjustment was done.

## 4. Results and Discussion

**Figure 5:**
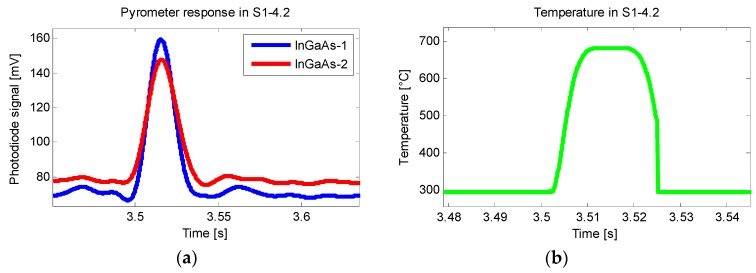
Photodiode signal (**a**) and resulting temperature (**b**).

In Figure 5, an example of one of the measurements can be seen in which the obtained signal from the pyrometer was cleared using a low pass filter. The two voltage signals, Figure 5a, represent the two cells that comprise each sensor. Combining the two signals and comparing the data with the calibration, a temperature signal is obtained Figure 5b. 

**Figure 6:**
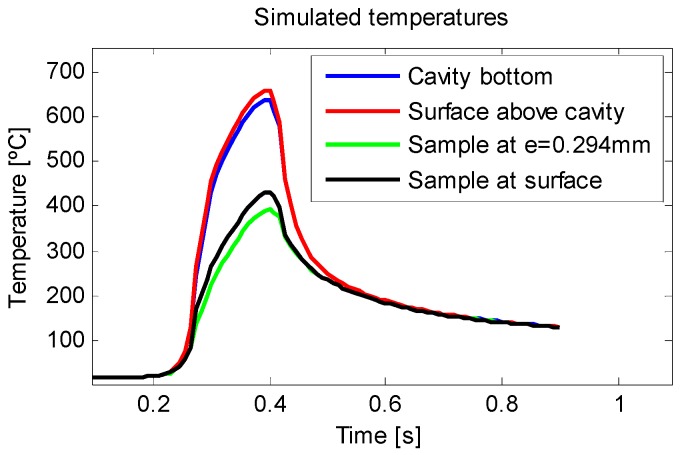
Simulated temperatures of different points of the model representing the temperature distortion caused by the cavity.

Using this data, the finite difference thermal model (described above) was fed. The temperature of four points of the model was monitored at each simulation. The blue line represents the bottom of the cavity and the red line represents the surface above. In addition, two more points were added to show the influence of the presence of a cavity on the workpiece. The green line represents a point that is at the same depth as the bottom of the cavity, which was not influenced by the presence of the cavity, whilst the black line represents the surface above this point. The progression in temperature of the four points during the course of the simulation period can be seen in Figure 6.

**Figure 7:**
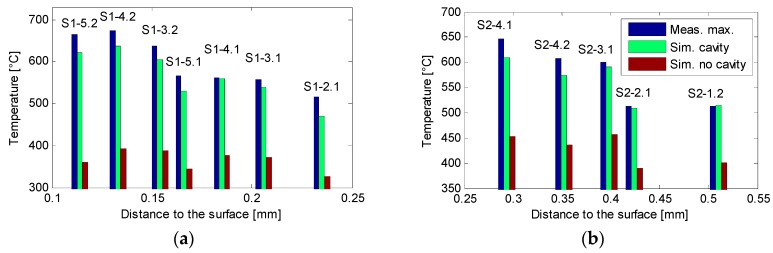
Maximum measured and simulated temperatures in the cavity in condition set S1 (**a**) and in S2 (**b**).

**Figure 8:**
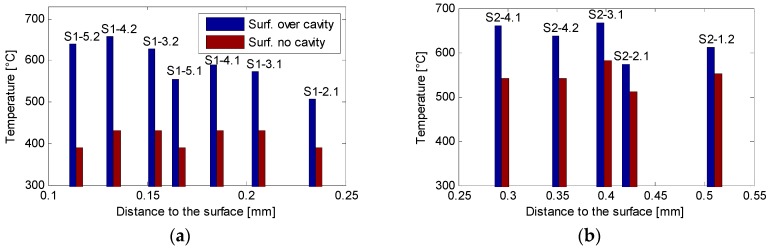
Maximum surface temperatures in condition set S1 (**a**) and in S2 (**b**).

The data gathered from the experiments and the corresponding simulations made for each test can be observed in Figure 7. Each of the graphs shows the maximum measured temperature on the tests of each cavity (Blue) paired with their corresponding maximum simulated temperature (Green) as well as the temperature of the same point, excluding the effect of the cavity (Red). Figure 8 presents the estimated surface temperatures over the cavity (Blue) and at the same point excluding the thermal distortion caused by the cavity (Red).

Figure 8 clearly shows the effect caused by the presence of the cavity, which is the subject of this work. It is clear that the measured and simulated temperature at the bottom of the cavity show a tendency to grow as the distance to the surface narrows, while the temperature at the same point, excluding the cavity, remains stable.

It is evident that the surface temperature estimation error caused by not considering the presence of the cavity was 19.7% when the distance to the ground surface was 0.51 mm on Test S2-1.2 (see Figure 8b). When the distance to the surface was narrower, this error became higher as the effect of the cavity amplifies. In S1-5.2, the error reached 63.4% at a distance of 0.114 mm (see Figure 8a). The progressive narrowing of the workpiece added to this effect, causing the temperature difference to be even higher. In short, the same amount of heat was being forced into a smaller volume and caused the temperature to rise.

The mean error in the measurements of the S1 tests was 5.4% in S1 and 8.4% in S2 if these are compared with the simulated bottom cavity temperatures. This estimation can be accepted as accurate given that the heat partition coefficient and real contact length were considered to be constant. This adjustment for each of the tests could be the focus of further experiments. This theoretical to experimental variation of parameters was also present in the h film coefficient factor of the cooling contour condition, both of which affected the temperature estimation of the model. This is the reason why the difference between the maximum measured temperature and the maximum simulated temperature varied between the tests.

## 5. Conclusions

The present work presents a novel temperature characterization system specifically designed for grinding processes. As expected, the obtained results demonstrated the necessity of combining the measurements with a thermal model that takes into account the effect of the cavity. Neglecting this effect adds an error of up to 70% over the measured temperature at 0.1 mm from the surface. This is unacceptable considering the volatility of the variables involved in the measurement of grinding temperatures. The results suggest that at larger distances from the surface the error is smaller. It is therefore necessary to argue that when surface temperatures are low the measurement distance must be reduced to precisely capture the temperature. This implies the use of the model, since the error, as stated, will grow when measurements are taken closer the surface. 

This innovative measurement system, combined with the specifically developed model, has been demonstrated to accurately estimate the surface temperature through measurement inside the cavity while taking into account any resulting thermal distortion. The temperature study presented in this work was clearly a first step in the thermal characterization of grinding processes. The design features of the pyrometer described in Section 2 make this device very useful for further research in the thermal aspects of grinding. The thermal model used in tandem with the pyrometer presented allows for a considerable number of thermal estimations to be made in various processes as well as in different grinding situations.

As mentioned, the heat partition for each case was considered to be constant and has emerged as an interesting subject for further investigation. The exact calculation of the heat partition in different conditions is perfectly within the scope of the thermal model and temperature measurement that were developed and described in this work.

## Figures and Tables

**Table 1:** Theoretical parameter Set 1 (S1) and Set 2 (S2).

Parameter	Set 1	Set 2
${Q’}_{w}$ (Removal rate) [mm^3^/(mm.s)]	6.67	6.67
$a_{e}$ (Depth of cut) [mm]	0.02	0.03
$v_{w}$ (Feed rate) [m/min]	2	1.334
$l_{\mathrm{cg}}$ (Geometric contact length) [mm]	2.47	3.03
Acquisition frequency [Hz]	9000	9000

**Table 2:** Real values of the grinding parameters measured on the tests.

Test Label	$a_{er}$ Real Depth of Cut [mm]	$v_{w}$ Feed Rate [m/min]	P Grinding Power [W]	e Distance to the Surface [mm]
S1-1.1	0.017	2.0	2923	0.274
S1-1.2	0.017	2.0	2923	0.222
S1-2.1	0.014	2.0	2871	0.235
S1-2.2	0.014	2.0	2871	0.183
S1-3.1	0.017	2.0	3078	0.206
S1-3.2	0.017	2.0	3078	0.154
S1-4.1	0.017	2.0	2957	0.185
S1-4.2	0.017	2.0	2957	0.133
S1-5.1	0.014	2.0	3111	0.166
S1-5.2	0.014	2.0	3111	0.114
S2-1.1	0.028	1.334	2834	0.452
S2-1.2	0.028	1.334	2834	0.51
S2-2.1	0.024	1.334	2816	0.424
S2-2.2	0.024	1.334	2816	0.482
S2-3.1	0.03	1.334	2858	0.398
S2-3.2	0.03	1.334	2858	0.456
S2-4.1	0.026	1.334	2835	0.294
S2-4.2	0.026	1.334	2835	0.352
